# Efficacy and safety of a subacromial continuous ropivacaine infusion for post-operative pain management following arthroscopic rotator cuff surgery: A protocol for a randomised double-blind placebo-controlled trial

**DOI:** 10.1186/1471-2474-9-56

**Published:** 2008-04-22

**Authors:** Jennifer A Coghlan, Andrew Forbes, Simon N Bell, Rachelle Buchbinder

**Affiliations:** 1 Monash Department of Clinical Epidemiology, Cabrini Hospital, Monash University, Melbourne, Australia; 2 Department of Epidemiology and Preventive Medicine, Monash University, Melbourne, Australia; 3 Department of Surgery, Monash University, Melbourne, Australia; 4 Melbourne Shoulder and Elbow Centre, Melbourne, Australia

## Abstract

**Background:**

Major shoulder surgery often results in severe post-operative pain and a variety of interventions have been developed in an attempt to address this. The continuous slow infusion of a local anaesthetic directly into the operative site has recently gained popularity but it is expensive and as yet there is little conclusive evidence that it provides additional benefits over other methods of post-operative pain management.

**Methods/Design:**

This will be a randomised, placebo-controlled trial involving 158 participants. Following diagnostic arthroscopy, all participants will undergo arthroscopic subacromial decompression with or without rotator cuff repair, all operations performed by a single surgeon. Participants, the surgeon, nurses caring for the patients and outcome assessors will be blinded to treatment allocation. All participants will receive a pre-incision bolus injection of 20 mls of ropivacaine 1% into the shoulder and an intra-operative intravenous bolus of parecoxib 40 mg. Using concealed allocation participants will be randomly assigned to active treatment (local anaesthetic ropivacaine 0.75%) or placebo (normal saline) administered continuously into the subacromial space by an elastomeric pump at 5 mls per hour post-operatively. Patient controlled opioid analgesia and oral analgesics will be available for breakthrough pain. Outcome assessment will be at 15, 30 and 60 minutes, 2, 4, 8, 12, 18 and 24 hours, and 2 or 4 months for decompression or decompression plus repair respectively.

The primary end point will be average pain at rest over the first 12-hour post-operative period on a verbal analogue pain score. Secondary end points will be average pain at rest over the second 12-hour post-operative period, maximal pain at rest over the first and second 12-hour periods, amount of rescue medication used, length of inpatient stay and incidence of post-operative adhesive capsulitis.

**Discussion:**

The results of this trial will contribute to evidence-based recommendations for the effectiveness of pain management modalities following arthroscopic rotator cuff surgery. If the local anaesthetic pain-buster provides no additional benefits over placebo then valuable resources can be put to better use in other ways.

**Trial registration:**

Australian Clinical Trials Register Number ACTR12606000195550

## Background

Surgical options for rotator cuff disease that has failed to improve with conservative treatments include open or arthroscopic subacromial decompression (ASD) [1] with or without rotator cuff repair (RCR) [2]. Arthroscopic approaches are being increasingly used because of purported advantages including earlier recovery, hypothesised to be due to preservation of the deltoid muscle with this approach; smaller scars; and the ability to access the glenohumeral joint to exclude other causes of shoulder pain. We recently completed a Cochrane systematic review of randomised controlled trials to determine the effectiveness and safety of surgery for rotator cuff disease [3]. We identified six trials that had compared arthroscopic to open subacromial decompression and while it was not possible to draw firm conclusions due to their overall poor quality, none of the trials reported significant differences between trial arms in terms of comparative improvements in pain, function or participant evaluation of success, while four trials reported earlier recovery with arthroscopic decompression. There were also no differences between trial arms for adverse events including post-operative adhesive capsulitis (or stiff painful shoulder or frozen shoulder).

Post-operative pain is often a problem following major shoulder surgery and may delay mobilisation of the shoulder [4]. It has been postulated that this contributes to the relatively high reported rates of post-operative adhesive capsulitis (10%) [1]. Optimal management of post-operative pain may therefore also be important in aiding early rehabilitation and reducing the incidence of post-operative adhesive capsulitis.

A variety of strategies may be used to manage post-operative pain including low dose continuous intravenous opioids with or without patient managed bolus doses using patient controlled analgesia (PCA) pumps [5], intra-articular injections of morphine or local anaesthetic [6], regional nerve blocks [7], non-steroidal anti-inflammatory drugs (NSAIDs) [8], and oral analgesics. While effective, there are known disadvantages to each of these modalities. Most PCA pump systems require expensive equipment [5] (around AU$3,000 at the hospitals in our study), as well as education of patients and monitoring by nursing staff. Strong opioid analgesia may cause nausea and vomiting, respiratory depression, sedation and constipation; neural blockade may be complicated by pneumothorax, nerve injury [9] and respiratory depression [10] while there are numerous potential adverse effects of NSAID therapy include peptic ulcer disease, platelet dysfunction, hypertension, fluid retention and renal impairment [11].

Use of a pre-emptive (before incision) bolus dose of local anaesthetic (e.g. lignocaine, bupivacaine or ropivacaine) injected into either the glenohumeral joint and/or subacromial space is gaining wide acceptance as an alternative method for pain management following shoulder surgery. Pre-emptive analgesia is purported to induce prophylactic neural blockade thus blocking nociceptive input and reducing post-operative pain [12]. The effect may be enhanced by the addition of a direct operative site infusion of local anaesthetic delivered by disposable infusion pumps. For example, elastomeric pumps provide direct pre-set delivery of local anaesthetic to the shoulder joint via a fine multi orifice slow infusion catheter which is easily positioned by the surgeon. The catheter is accurately placed in the subacromial space under arthroscopic or direct visualisation, reducing the risk of nerve, soft tissue and vascular damage [13].

Administration of local anaesthetic has been performed by direct infiltration, intermittent lavage, continuous infusion with PCA bolus or PCA alone and some of the trials have also included pre-emptive or intra-operative analgesia and/or NSAIDS. Shoulder operations have included open as well as arthroscopic surgery, and surgery to the capsule and ligaments as well as to the rotator cuff. Seven previous randomised placebo-controlled trials of differing methodological quality have assessed the efficacy of local anaesthetic (lignocaine, bupivacaine or ropivacaine) continuously infused into the operative site following various types of major shoulder surgery [13-19]. While difficult to make direct comparisons between trials because of these differences, some trials have found that compared with placebo, local anaesthetic resulted in lower post-operative pain scores with or without less analgesia use, [14,15,17-19] while other studies have found no significant differences in regard to pain and use of rescue medication between treatment groups [13,16].

While ropivacaine is now considered the local anaesthetic of choice despite its greater cost, earlier trials used either the shorter acting lignocaine or bupivacaine. Bupivacaine has been shown to cause greater dose related central nervous system and cardiac toxicity than ropivacaine [20]. Ropivacaine has less motor blockade than either of these drugs [21], which may be clinically important in terms of active post-operative mobilisation of the shoulder. Previous trials have also failed to take into account potential confounding due to different operative procedures or, in the case of multiple surgeons and/or centres, potentially different surgical skills, technique, equipment and peri-operative and post-operative care.

In our setting, post-operative pain management has traditionally consisted of pre-emptive local anaesthetic (ropivacaine) and post-operative PCA opioids. Recently and concurrently, both intra-operative intravenous parecoxib a selective COX-II inhibitor, and direct operative ropivacaine infusion pumps were introduced as standard practice although evidence for their added benefits is limited. The disposable pump with single or dual catheters and ropivacaine costs $AUD300–600 and $155 respectively (at time of publication), while a single pre-emptive bolus of 20 mls 1% ropivacaine costs $20 and an IV bolus of parecoxib (40 mg) costs only $25. Reported adverse effects of infusion pumps include difficulty in removal of the catheter, too rapid delivery of the drug, leakage with local contamination, and breakage of the catheter tip leaving a remnant in the joint [22,23]. To warrant continued use of the ropivacaine infusion, it would need to be demonstrated that its benefit/s in terms of pain relief, reduction in drug-related adverse effects, earlier shoulder mobilisation and/or reduced hospital stay is/are worth the additional costs.

The aim of this randomised placebo controlled trial is to determine the efficacy and safety of a continuous infusion of ropivacaine 0.75% (Naropin – AstraZeneca) delivered by a single catheter 'Painbuster'(I-Flow Corporation, Lake Forest, USA) pain management system when used following elective arthroscopic rotator cuff surgery. Our hypothesis is that continuous infusion of local anaesthetic following rotator cuff surgery would be equivalent to pain relief provided by a placebo in the setting of pre-emptive ropivacaine, intra-operative parecoxib and post-operative intravenous morphine and oral dextropropoxyphene hydrochloride/paracetamol available on demand.

## Methods

### Design

**Figure 1:**
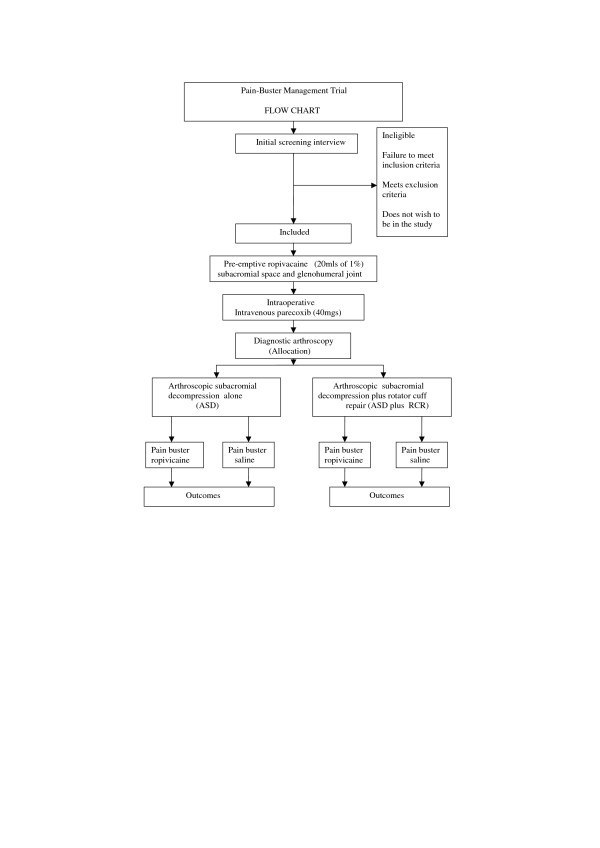
Trial profile

This will be a randomised placebo-controlled trial (Figure 1). Participants, the single surgeon, nurses caring for the participants and outcome assessors will all be blinded to treatment allocation.

### Setting and Participants

Participants will be recruited from the private community-based practice of a single orthopaedic shoulder surgeon in Melbourne, Australia. The decision to include only one surgeon will eliminate any potential confounding of results due to differences in surgical skill, technique and care across treatment arms. One of two elective operations will be performed based upon diagnostic arthroscopy at the time of operation: ASD or ASD plus RCR. All rotator cuff surgery will be performed at either the Mercy Private Hospital or Linacre Private Hospital in Melbourne, Australia.

All consecutive, eligible patients who fulfil selection criteria will be offered study participation. Inclusion criteria will be adults aged 18 years or over in whom elective subacromial decompression or rotator cuff repair is planned. Exclusion criteria will be: (i) patients with massive or irreparable rotator cuff tears; (ii) patients requiring grafts to fill the defect; (iii) previous injury or surgery to the shoulder i.e. fracture or revision surgery; (iv) previous mastectomy on the affected side; (v) peripheral neuropathy affecting the upper limbs; (vi) chronic opioid use; (vii) morbid obesity (body mass index greater than 35 kg per metre squared); (viii) Parkinson's disease; (ix) pregnancy; (x) contraindication to parecoxib including aspirin sensitive asthma, history of recent gastric or duodenal bleeding, severe renal impairment or sulphonamide allergy; (xi) inability to understand written and/or spoken English.

### Ethics

The Monash University and Mercy Aged and Health Care Ethics Committees have approved the study and all participants will provide written informed consent.

### Randomisation

Once eligibility has been assessed following diagnostic arthroscopy, participants will be randomly assigned in random permuted blocks of differing sizes to either active treatment (ropivacaine) or placebo (normal saline), stratified by type of operation (ASD or ASD plus RCR) (Figure 1). The randomisation sequence will be generated using a computer generated table of random numbers by the study biostatistician. Stratified allocations will be sealed in opaque and consecutively numbered envelopes kept in a locked location. These will be opened in sequence by an independent administrator not involved in the eligibility or outcome assessment or treatment administration.

### Interventions

The surgeon's standard surgical protocol for rotator cuff surgery will be adhered to as follows: Participants will be asked to cease all NSAIDs two weeks prior to commencement of the study but will be allowed codeine, paracetamol or dextropropoxyphene. All operations will be performed under general anaesthesia using a standardised method consisting of propofol 2–2.5 mg/kg and fentanyl 1–2 mcg/kg as induction agents, nitrous oxide/oxygen 2:1, +/- isoflurane, morphine as required to maintain heart rate, blood pressure, respiratory rate within normal limits, and spontaneous ventilation on a laryngeal mask unless contra-indicated.

All patients will receive a total of 20 mls of 1% ropivacaine injected as a pre-emptive bolus dose into both the glenohumeral joint and the subacromial space fifteen minutes prior to insertion of the arthroscope. During the operation all patients will receive an intravenous bolus of 40 mg of parecoxib. At the conclusion of the surgery, all patients will have insertion of a 'Pain buster' catheter into the subacromial space with the position verified by direct or arthroscopic vision. According to blinded randomised treatment allocation the 'Pain buster' elastomeric pump will contain 180 mls of either 0.75% ropivacaine or an identical placebo of normal saline administered at 5 mls per hour.

All patients will have access to alternative analgesia for break through pain including both a PCA pump of intravenous morphine available on demand with a lock out period and dextropropoxyphene hydrochloride/paracetamol. All medications will be recorded.

### Outcome assessment

All assessors collecting and assessing outcome will be blinded to treatment allocation. One assessor will perform all baseline, and 2 or 4 month (JC) assessments. Post operative assessment of pain, analgesia and duration of hospital stay will be recorded by nursing staff.

Baseline data will include gender, age at operation, dominance, side of operation, height, weight, history of diabetes, employment, duration of symptoms and compensation status. As a verbal analogue pain scale (VAPS) will be used to measure pain postoperatively, participants will be taught to use the scale at the time of pre-operative baseline assessment to measure current level of pain at rest. The VAPS is an 11-point scale where 0 = no pain and 10 = worst pain imaginable.

Outcomes will be assessed post operatively at 15, 30 and 60 minutes, 2, 4, 8, 12, 18 and 24 hours, and 2 or 4 months for ASD or ASD and RCR respectively. Average pain at rest and maximal pain over the first and second 12 hour post-operative periods, will be computed using the VAPS. The amount of rescue medication used over the first and second 12 hour post-operative periods will be computed as intravenous opioid intake in mgs and oral analgesic intake as a tablet count.

Range of movement of the shoulder will be measured at baseline and at 2 or 4 months for ASD or ASD plus RCR respectively. Active shoulder movements will be measured according to a standardised and reliable protocol [24]: Total shoulder flexion (TSF), total shoulder abduction (TSA) and external rotation in neutral (ERN) will be measured with a gravity inclinometer (in degrees) and internal rotation will be assessed by measuring distance (in centimetres) from the base of the occiput to how high the hand will reach up behind the back (HBB). Post-operative adhesive capsulitis will be defined as having symptoms of shoulder pain and stiffness and restriction of movement of at least 30° in two or more planes at 2 or 4 months post-operatively for ASD or ASD plus RCR respectively.

Length of hospital stay will be measured in days from time of surgery and all adverse effects will be recorded.

The primary end point will be the average pain at rest over the first 12 hour post-operative period. Secondary end points will be the average pain at rest over the second 12-hour post-operative period, the maximal pain over the first and second 12-hour periods, the amount of rescue medication used, late discharge (>1 bed day) from hospital following ASD, early or late discharge (< or > 2 bed days) following ASD plus RCR, and the incidence of post-operative adhesive capsulitis up to 2 or 4 months following ASD or ASD plus RCR respectively.

### Sample Size

Sample size is based upon clinical equivalence being deemed as a difference in mean pain scores of +/-1 point on a 10 point scale. That is, any difference between groups of 1 point or less will be regarded as equivalent for practical purposes. Statistically, equivalence will be declared if the 90% CI for the difference in mean pain scores between the groups lies entirely within the interval -1 to +1. To have 80% probability that this equivalence will occur when the two infusion therapies are actually exactly equivalent requires 70 patients per arm, assuming a standard deviation of 2 points within each arm (obtained from pilot work). This sample size will also be able to detect a difference of 0.95 points with 80% power and a two-sided 5% significance level. Allowing for 10% dropout, the sample size is inflated to 78 patients per group.

### Data Analysis

The principal analyses will involve the comparison of average scores of pain at rest in the first 12 hours post-operation between the ropivacaine pump infusion and placebo patients using an intention-to-treat analysis. This will be performed by multiple regression adjusting for infusion type and type of operation. Similar analyses will compare maximal pain at rest in the first 12 hours and average and maximal pain at rest in the second 12-hour post-operative period. Sensitivity analyses to assess dependence upon potential baseline imbalance in prognostic factors will also be performed. Other secondary outcome analyses will include amount of morphine administered, the incidence of complications, late discharge from hospital (>1 bed day) following ASD, early or late discharge (< or > 2 bed days) following RCR, and the incidence of post-operative adhesive capsulitis up to 2 or 4 months following ASD or ASD plus RCR respectively. Further analyses will assess differences between arms in maximum and average pain scores at rest adjusted for amount of opioid used.

## Discussion

The results of this trial will contribute to evidence-based recommendations for the effectiveness of pain management modalities following arthroscopic rotator cuff surgery. If we can show that the pain-buster is equivalent to current treatment and adds nothing to current pain management then this will reduce the costs of the surgical procedure and hospitalisation. Strengths of our trial include blinding of all personnel involved in participant care including the surgeon; assurance of homogeneity of study population by pre-randomisation identification of the surgical procedure required; use of a realistic placebo infusion; use of a single surgeon and single post operative regime limiting confounding; and statistical power to detect small differences of importance.

## Competing interests

The author(s) declares that they have no competing interests.

## Authors' contributions

JC, RB, AF and SB conceived and designed the trial protocol. AF designed the statistical analysis. JC, RB and AF drafted the manuscript. All authors read and approved the final manuscript.

## Pre-publication history

The pre-publication history for this paper can be accessed here:


